# High Performance PA 6/Cellulose Nanocomposites in the Interest of Industrial Scale Melt Processing

**DOI:** 10.3390/polym13091495

**Published:** 2021-05-06

**Authors:** Pruthvi K. Sridhara, Fabiola Vilaseca

**Affiliations:** Advanced Biomaterials and Nanotechnology, Department of Chemical Engineering, University of Girona, 17003 Girona, Spain; pruthvi.sridhara@udg.edu

**Keywords:** polyamide 6, cellulose nanofibers, nanocomposites, melt processing, mechanical properties, thermal properties

## Abstract

On an industrial scale, it is a challenge to achieve cellulose based nanocomposites due to dispersion issues and high process temperatures sensitivity. The current study describes methods to develop mechanically strong and thermally stable polyamide 6 (PA 6) and cellulose nanofibers (CNF) composites capable of tolerating high processing temperatures. With PA 6 being a very technical polymer matrix to be reinforced with CNF, good dispersion can be achieved with a high speed kinetic mixer and also shield the CNF from excess thermal degradation by implementing extremely short processing time. This paper presents an industrially feasible method to produce PA 6/CNF nanocomposites with high CNF composition processed by a high speed kinetic mixer (GELIMAT^®^) followed by compression molding to obtain a homogenous and thermally stable nanocomposites aimed at high performance applications. PA 6 was reinforced with three different wt % formulations (5, 15 and 25 wt %) of cellulose nanofibers. The resulting nanocomposites exhibited significant increase in Young’s modulus and ultimate strength with CNF content, owing to the effective melt processing and the surface charge density of the CNF, which necessitated the dispersion. The thermal stability and polymer crystallinity with respect to CNF composition for the PA 6/CNF nanocomposites were examined by TGA and DSC analysis. Rheology studies indicated that viscosity of the composites increased with increase in CNF composition. Overall, this work demonstrates industrially viable manufacturing processes to fabricate high performance PA 6/CNF nanocomposites.

## 1. Introduction

Polyamide 6/nylon 6 (PA 6) is an engineering thermoplastic boasting good mechanical integrity, thermal properties, chemical and dimensional stability. In recent years, composites of PA 6 have gained tremendous interest in high performance and light weight replacement for metals and rubbers in the industrial sector especially in the automotive industry [1]. The performance of PA 6 can be further enhanced by reinforcing the polymer matrix with fibrous or crystalline filler [2]. Industrial interest has shifted towards eco-friendly and economic means to produce greener materials to reduce the impact of carbon footprint on the environment. Incorporating natural fibers to polymeric matrixes not only generates lower CO_2_ emission, but also increases the biodegradability of the material [3]. Commonly used synthetic fibers such as glass and carbon fibers possess higher density than natural fibers, comparably to such synthetic fibers, natural fibers reinforcement can provide high specific mechanical properties to the polymer composites [4]. Further studies have revealed that incorporating natural fiber-fillers with their dimension in nanoscale, significantly improves the mechanical properties of neat polymers due to their high specific strength and high aspect ratio [5].

With cellulose being one of the most abundant natural polymer, reinforcing PA 6 with cellulosic nanofillers is an effective way to develop new composites, further widening the potential of applications for polymeric composites [6]. There are two major families among nanocellulose: cellulose nanocrystals (CNC) and cellulose nanofibers (CNF). CNF are easily produced with high yield when compared to CNCs and, generally, CNF are more thermally stable than CNC when they are purified thoroughly from lignin and hemicellulose, which are thermally volatile constituents [7]. Enzymatically treatment of cellulose is one of the simplest and ecofriendly ways to defibrillate and extract CNF [8]. These CNF embody a swirled and flexible characteristic, and typically having their lengths in the range of 0.7–2 µm and diameters of around 5–30 nm, with majority of nanofibers with diameters within 15–30 nm [9,10]. As PA 6 and CNF are polar materials and are hydrophilic in nature, good interaction is formed between them making them compatible with each other [11].

CNF have a young’s modulus of 24 GPa and tensile strength of 320 MPa [12]. These mechanical properties can be imparted to the end-use composite by dispersing them into polymer matrix by various processing methods. However, there are several challenges in reinforcing CNF with PA 6 matrix given the processing temperatures of PA 6 are close to the degradation onset temperatures of the CNF. On an industrial scale, it is important to optimize processing methods and modify materials if necessary, to produce high performance nanocomposites and to improve the scalability of the overall production. While processes like solvent casting have been successful in negating the degradation effect by not involving high temperatures, solvent casting process is most suited to small-scale or lab conditions and besides, extensive use of solvents generates chemical wastes making it not ecofriendly [13,14]. Conventional methods like melt processing, injection molding and compressing molding have already been well established in the current day industry. To this day, many researchers have been successful in melt processing cellulose composites with polyethylene (PE), polypropylene (PP), polyvinyl alcohol (PVA) and polylactic acid (PLA) [15]. Additionally, previous works within our group have successfully melt processed PA 6 composites with well dispersed microfibrillated/pulp fibers with improved mechanical properties and without compromising on thermal stability [16,17].

**Figure 1:**
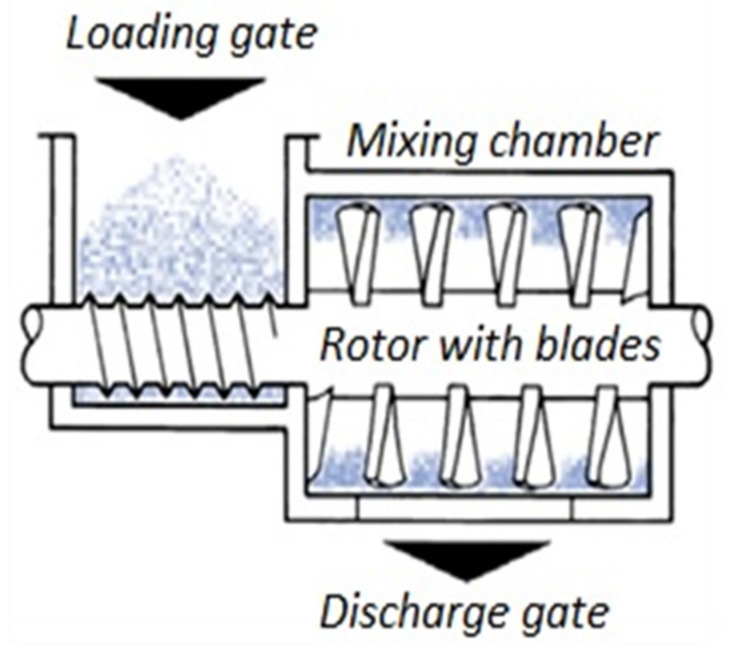
Schematic of GELIMAT^®^ feeding and mixing chamber [18].

The obstacles of thermal stability can be overcome and good dispersion of CNF within the PA 6 matrix can be achieved by melt compounding with a high speed thermo-kinetic mixer such as a GELIMAT^®^. The schematic of a Gelimat mixing chamber is shown in Figure 1 [18]. The process involves exerting high shear rates on the materials, which increase the temperature of the compounding mixture and a final homogenous compound with excellent dispersion can be obtained [19]. Materials can be compounded in extremely less time (<60 s), therefore, the CNF are exposed to processing temperatures for a shorter period, thus, preserving thermal stability. Compared to twin screw extruder and Brabender^®^, the Gelimat produces well dispersed and distributed composites [20]. Moreover, homogenous compounds can be obtained in a single cycle, whereas, similar levels of dispersion require two or three passes in an extruder. In our study, we push forward the scalable industrial method to produce high performance PA 6/CNF nanocomposites with high weight fraction of CNF. To further improve scalability and due to the matrix–nanofiber interface, no coupling and/or dispersing agent was used, which was used in some studies to improve processability [21]. Nanocomposite samples were produced using compression molding and characterized for tensile and thermal properties. Rheological behavior and water absorption was analyzed with the interest for industrial scale processing methods.

## 2. Materials and Methods

**Figure 2:**
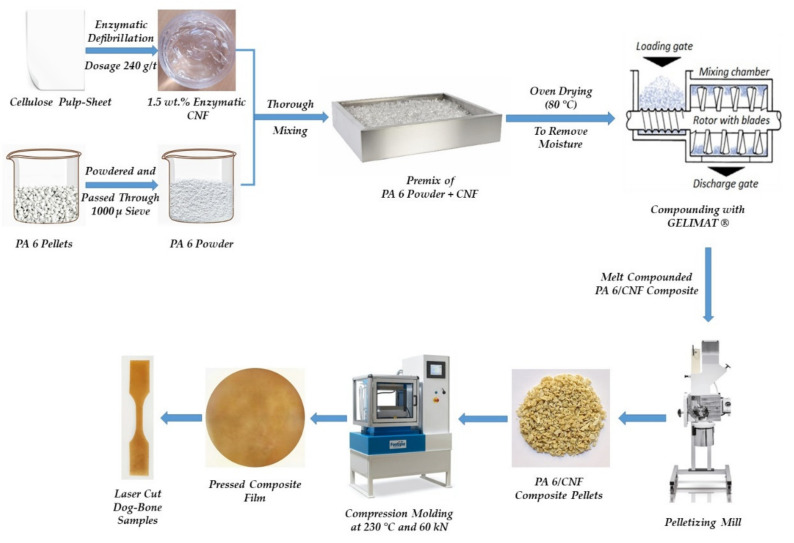
Schematic representaion of the manufacture process for PA 6/CNF nanocomposites [18,22,23].

The PA 6 (density ρ = 1.14 g/cm^3^) was obtained commercially in pellet form from Radici Plastics Iberica SL (Barcelona, Spain). The PA 6 pellets were then powdered from Powder Plastics Europe SL (Valls, Spain) and the powdered PA 6 was passed through a 1000-micron sieve. The major particle size distribution of the powdered PA 6 ranged between 150 and 800 microns. For the rest of this paper, powdered PA 6 will be denoted as just PA 6. Cellulose was originally obtained in form of pulp sheets from Domsjö Fabriker (Örnsköldsvik, Sweden). These sheets were of high cellulose content (>97% pure cellulose) primarily derived from softwood. To prepare the CNF, an endoglucanase enzyme namely Novozyme 476 obtained from Novozymes^®^ AS (Copenhagen, Denmark) was utilized to expedite the disintegration of cellulose pulp. The Schematic of the complete composites preparation process is depicted in Figure 2.

### 2.1. Processing of PA 6/CNF Nanocomposites

#### 2.1.1. Preparation of CNF

The cellulose pulp was subjected to enzymatic treatment/disintegration as described by Heriksson et al. [8]. Initially, the cellulose pulp was broken up in distilled water and was amalgamated in a disintegrator (Papelquimia SA, Besalu, Spain) to form 3 wt % cellulose pulp fibers suspension. The cellulose pulp fibers were then mechanically subjected to a PFI mill (IDM Test SL, San Sebastian, Spain) at 1000 revolutions to increase the fibers swelling in water and provide adequate accessibility for the enzyme. The enzymatic treatment was carried out by dispersing the 3 wt % pulp fiber in 50 mM tris(hydroxymethyl)aminomethane/HCl buffer with pH 7 and 0.02 wt % enzyme. The fibers were incubated for 2 h at 50 °C and later washed on a Büchner funnel. This was followed by keeping the fibers at 80 °C for 30 min to stop the enzyme activity and was washed again. Additionally, the fibers were again subjected to the PFI mill at 4000 revolutions. After the enzyme treatment, 1.5 wt % of pretreated fibers solution in distilled water was prepared and subjected to homogenization (Panda Plus, GEA Niro Soavi, Parma, Italy). The fibers were passed through the homogenizer 6 times and a consistent CNF gel was procured.

#### 2.1.2. Melt Compounding

In order to prepare 5 wt %, 15 wt % and 25 wt % of PA 6/CNF nanocomposites, batches of PA 6 respectively were dried for 8 h in an 80 °C oven to remove any moisture content. Each batch of dry PA 6 was thoroughly mixed with the respective amount of CNF gel to obtain the corresponding CNF wt % formulation of the nanocomposite. These mixtures were then dried in a 60 °C oven until all the gel-moisture content was removed. The dry PA 6/CNF mixture was introduced into the Gelimat^®^ (Draiswerke G5 S, Ramsey, NJ, USA) at a rotor speed of 300 rpm. The loading gate was shut and the rotor speed was increased to 2500 rpm. The action of the blades at such high speed allowed a mixture to reach melting temperature (220–230 °C) in less than 20 s. The completion of the compounding process was signified by the drop in current drawn by the rotor. Simultaneously, the discharge gate was opened and the compounded mixture was collected and cooled immediately using a cold bath. The compounding process was repeated to obtain the remaining formulations of the nanocomposites. Further, the compounded composite mixtures for all the formulations were produced into composite pellets respectively using a pelletizer (SM100, Retsch GmbH, Haan, Germany).

### 2.2. Characterization of PA 6/CNF Nanocomposites

#### 2.2.1. Compression Molding

All the formulations of nanocomposites pellets obtained from the compounding process was compression molded into films using a laboratory hydraulic hot press (Fontijne Presses BV, Delft, The Netherlands) at 230 °C under a pressure of 60 kN for 10 min. Spacers with 0.5 mm thickness were positioned to control the thickness of the produced films. The obtained films were light brown in color and translucent with no optical signs of CNF aggregation. However, minute aggregation was observed in the 25 wt % formulation.

#### 2.2.2. Mechanical Testing

PA 6/CNF nanocomposites were tensile tested using a 5 kN Instron^®^ Type 1122 Test (Norwood, MA, USA) machine. Dog-bone shaped samples from the PA 6/CNF nanocomposites films were cut using a die as per ASTM D638 (Type V) specifications. The pressed dog-bone samples were tested under two parameters. Firstly, dry samples, where the dog-bone samples were dried in an 80 °C oven for 6 h prior to testing. Secondly, 48 h conditioned samples, where the samples were placed in a climatic chamber at 23 ± 2 °C and 50% ± 5% relative humidity for 48 h (ASTM D618 13) prior to testing. Tests were carried out at room temperature at a strain rate of 2 mm min^−1^ and a gauge length of 30 mm. The thickness and width of the narrow section for each sample was measured using a digital micrometer (Starrett^®^, Athol, MA, USA). Trials were repeated to establish statistical significance.

#### 2.2.3. Conductometric and Polyelectrolyte Titration of CNF

The content of carboxyl groups of the CNFs was determined by conductometric titration as described by Saito et al. [22]. CNF was dispersed rigorously in deionized H_2_O and 0.01 M HCl (Sigma Aldrich, Munich, Germany). The suspension was titrated with 0.5 M NaOH (Sigma Aldrich, Munich, Germany). Titration conductivity values were plotted against volume of NaOH added. The carboxyl content *S* (µMol/g) was calculated using Equation (1).

(1) $$S=\frac{V_{NaOH}\;\cdot \;C_{NaOH}}{W_{CNF}}\;\cdot 10^{6}$$

where *V_NaOH_* is the added volume of NaOH, *C_NaOH_* is the concentration of NaOH solution and *W_CNF_* is the weight of dispersed CNF.

The surface charge of CNF was also characterized for cationic demand, which was determined by polyelectrolyte titration using a Mütek PCD04 (BTG SL, UK) charger analyzer. The titration was performed through surface adsorption of diallyldimethylammonium chloride, poly-DADMAC, (Sigma Aldrich, Munich, Germany, molecular weight: 107 kDa) and the excess was titrated with polyethenesodiumsulphonate, PES-Na, (BTG, Éclépens, Switzerland) an anionic standard polymer.

#### 2.2.4. Rheology Study

The rheological behavior was studied using a melt flow indexer/MFI-type device (Ceast, Pianezza, Italy). Neat PA 6 and the compounded nanocomposite pellets were tested for MFI at 230 °C through a capillary (die) of specific diameter and length by pressure applied through dead weight *M* (kg) as per ASTM 1238-73. The geometrical dimensions of the MFI apparatus are: radius of the die *r* = 1 mm; radius of the heating cylinder *R* = 4.75 mm; length of the heating cylinder *L* = 30 mm (ISO 1133). The mass series was as follows: *M1–M2–M3......M7* (kg) = 1.2–2.16–3.8–5.0–7.16–10.0–12.16. For each mass used, five MFI values were measured for statistical significance.

Capillary rheological properties can be illustrated on a rheogram, which represents variation of dynamic viscosity *μ* (Pa s) versus shear rate *γ* (s^−1^). To go from *MFI* (g/10 min) to viscosity, the equations governing the flow of fluids inside a capillary (die) was used [23].

The apparent shear rate γ depends on the volume flow rate *Q* (m^3^s^−1^) and die radius *r* (m).

(2) $$\gamma =\frac{4\;\cdot \;Q}{\pi \;\cdot \;r^{3}}$$

The volume flow rate *Q* can be calculated using the MFI data and the respective hot density *ρ* (g cm^−3^) of the samples.

(3) $$Q=\frac{600\;\cdot \;MFI}{\rho \;\cdot \;t}$$

Hence,

(4) $$\gamma =\frac{2400\;\cdot \;MFI}{\pi \;\cdot \;r^{3}\;\cdot \;\rho \;\cdot \;t}$$

The viscosity μ is defined as shear stress *τ* (Pa) divided by the shear rate γ. The shear stress τ depend on the pressure *P* exerted at die inlet, i.e., the force *F* exerted by the mass *M* placed on the MFI piston.

(5) $$P=\frac{F}{\pi \;\cdot \;R^{2}}=\frac{M\;\cdot \;g}{\pi \;\cdot \;R^{2}}$$

Hence,

(6) $$\tau =\frac{P\;\cdot \;F}{2\;\cdot \;L}=\frac{M\;\cdot \;g\;\cdot \;r}{\pi \;\cdot \;R^{2}\;\cdot \;2\;\cdot \;L}$$

The above Equations (4) and (6) are for Newtonian fluids. PA 6 and respective composite blends have non-Newtonian behavior. Therefore, the apparent shear rates were corrected using the Rabinowitsch shear rate correction [24]. A first realistic approach to the rheological behavior was presented by the power law symbolized by Oswald law (Equation (7)).

(7) $$\tau_{T}=k\;\cdot \;\gamma_{T}{}^{n}$$

where $\tau_{T}$ is the true shear stress, *k* is a constant of the fluid and *n* is the flow index (pseudoplasticity index). To find out *n*, the curve Ln *τ* = f (Ln *γ*) was plotted from the calculated apparent stresses and shear rates obtained from the MFI data. With *n* being the slope and Ln *k* the intercept, the necessary Rabinowitsch correction was performed for the apparent shear rate *γ* (Equation (8)).

(8) $$\gamma_{T}=\frac{\left(3n+1\right)}{4n}\;\cdot \;\gamma \;$$

where $\gamma_{T}$ is the true shear rate, from which the true effective viscosity *μ* was calculated. Thus, the rheogram representing the rheological behavior of PA 6 and PA 6/CNF nanocomposites at 230 °C was obtained.

#### 2.2.5. Water Absorption Study

Three dog-bone samples for each formulation were chosen to conduct periodic mass measurements when submerged in water over a 24-h period. The dry samples were weighed before placing them in distilled H_2_O at room temperature. The samples were removed from the water and patted dry with Kimwipes^®^ prior to weighing. Mass measurements were taken at 0 h, 0.5 h, 1 h, 3 h, 6 h, 12 h, 18 h and 24 h. The weight percentage change was calculated using Equation (9), where *W_wet_* is the weight of sample after immersed in water and *W_dry_* is the dry weight of the sample.

(9) $$percent\;change=\frac{\left(W_{wet}-W_{dry}\right)}{W_{dry}}\cdot \;100$$

The samples were allowed to gain mass until they reached an equilibrium state. The water uptake kinetics can be modelled analytically by using Fick’s theory of dispersion. The diffusion coefficient *D* (m^2^s^−1^) of the nanocomposite samples with respect to the ability of moisture/water to penetrate the samples was deduced from Fick’s law. The diffusion coefficient was determined for shorter immersion time and thus, Fick’s law is stated as in Equation (10). Where $M_{t}$ (%) is the water uptake at a lower immersion time, $M_{\infty }$ (%) is the maximum mass gained, *L* (m) is the thickness of the sample and time *t* (s).

(10) $$\frac{M_{t}}{M_{\infty }}=2\;\cdot \;\left(\sqrt{\frac{D\;t}{\pi \;L^{2}}}\;\right)$$

#### 2.2.6. Thermal Properties

Thermogravimetric analysis (TGA) was conducted using a Mettler Toledo TGA 851 equipment (Greifensee, Switzerland). The temperature ranged from 25 to 600 °C with a 10 °C/min heating rate. The tests were carried out by placing the samples in an open platinum pan within a nitrogen environment. All the results were recorded using the STAR^e^ thermal analysis software. Additionally, TGA at isothermal conditions (230 °C) for the CNF gel was performed to check for thermal stability of CNF at processing temperatures.

In addition to TGA, a differential scanning calorimeter (DSC) was performed with DSC Q2000 equipment (TA Instruments, New Castle, DE, USA). The DSC was run three times for each formulation and the dry samples ranging from 6 to 8 mg were placed in aluminum pans. The samples underwent a heating cycle from 30 to 260 °C with a heating rate of 10 °C/min. All the results were reported by a TA Universal Analysis software. The degree of crystallinity ($\chi_{c}$) was calculated corresponding to the enthalpy of the melting endotherm using Equation (12), where $\Delta H_{sample}$ is the enthalpy of the melting endotherm of the sample from the heating cycle, $\Delta H_{polymer}$ is the melting enthalpy of PA 6 polymer in the sample, $w_{CNF}$ is the weight fraction of the CNF in the sample and $\Delta H_{100\%}$ is the theoretical melting enthalpy of 100% crystalline PA6, which is equivalent to 230 J/g [25,26].

(11) $$\Delta H_{polymer}=\Delta H_{sample}\;\cdot \;\left(\frac{1}{1-\frac{w_{CNF}}{100}}\right)$$

(12) $$\chi_{c}\;\left(\%\right)=\frac{\Delta H_{polymer}}{\Delta H_{100\%}}\;\cdot \;100$$

#### 2.2.7. Scanning Electron Microscopy (SEM)

The morphology of fractured samples due to elongation from tensile testing was characterized under a S4800 electron microscope (Hitachi, Ibaraki, Japan). The 5 wt % and 25 wt % formulations of the PA 6/CNF nanocomposites were analyzed at an accelerated voltage of 15 kV to distinguish CNF and PA 6 matrix. The fractured surfaces were coated with a thin layer of gold with the help of a sputter prior to observation.

## 3. Results and Discussion

**Figure 3:**
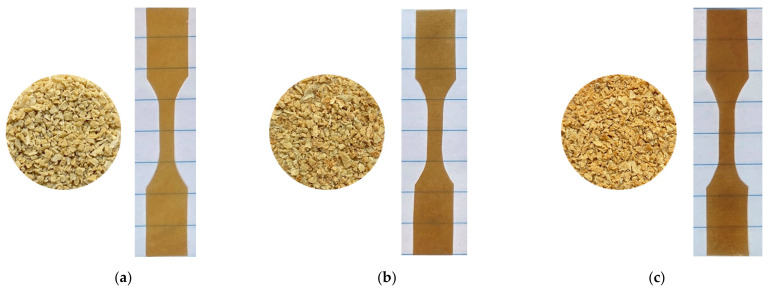
PA 6/CNF nanocomposites pellets and dog-bone samples for (**a**) 5 wt %, (**b**) 15 wt % and (**c**) 25 wt % formulations.

Samples were made for three different formulations of CNF composition: 5 wt %, 15 wt % and 25 wt %. The PA 6/CNF pellets and the respective homogeneous films obtained by Gelimat compounding and compression molding are shown in Figure 3. The processes resulted in a color change of slight brownish color and the films showed no optical aggregation of CNF. This discoloration of the pressed composite samples was expected due to the onset thermal degradation temperature of the CNF. This degradation is slow, mild and is mainly attributed to the dehydration of composite and formation of peroxides, which may catalyze the cellulose degradation [27,28]. However, our previous experience with pulp fiber composites suggest that it does not affect the mechanical integrity [16].

Mechanical properties of nanocomposites depend on many parameters like weight fraction of CNF, degree of dispersion of CNF and the size of the nanofibers. The mechanical behavior of the PA 6/CNF nanocomposites increased with the increase in CNF content with respect to neat PA 6. The best result for ultimate tensile strength (77.7 MPa) and modulus (5.6 GPa) was obtained for the 25 wt % formulation. All the data from the tensile tests are summarized in Table 1.

The improvement in the mechanical properties are directly correlated to dispersion and distribution of CNF within the PA 6 matrix [29]. Additionally, the processing methods did not compromise on the thermal stability and the melt compounding improved the homogeneity of the nanocomposites. The compatibility of CNF-PA 6 and the direct melt mixing of the composites allowed us to suppress the use any coupling agent and dispersive agent. This increases the scalability of the entire process, which is important at an industrial level. The reduction of deformation (strain) at break for the nanocomposites indicated good wetting and interaction between the CNF and PA 6 matrix, which allowed effective stress transfer and improved the stiffness [30]. Some studies on polyamide cellulose composites indicated improvement in mechanical properties for lower nanocellulose concentrations, but for higher concentration the tensile properties diminished. This could be due to excessive nanofiller–nanofiller interaction leading to poor reinforcement and embrittlement of the overall sample [31]. Another reason could be that increased filler content makes the polymer difficult to penetrate the space between the nanofillers resulting in poor wetting and hindering stress transfer through the interface [32]. Overall, for such high fiber weight fraction nanocomposites, the CNF exists as a 3D percolated network due to the hydroxyl groups on CNF resulting in hydrogen bonding between them [33]. Our findings support the fact that high performance, high cellulose weight fraction composites can be obtained by optimizing processing methods, which means our processing method was successful in penetrating PA 6 in between the percolated network of CNF. Hence, is accountable for the improvement in mechanical properties of the nanocomposites.

The enhanced mechanical properties are directly related to how well the CNF is dispersed within the PA 6 matrix. The dispersibility is indeed dependent on the surface charge density of the CNF, which governs the electrostatic repulsion between the individual fibers [34]. Polyelectrolyte and conductimetric titration revealed the cationic demand of 258 µeq. g/g and the carboxyl group content of 54 µmol COOH/g, respectively, for the current enzymatic CNF. This suggests that amount of electrostatic repulsion between the CNFs is sufficient to be well dispersed within the PA 6 matrix, which is further corroborated by improvement in tensile properties [35]. Nevertheless, shorter chained polyamides such as PA 6 possess higher frequency of amide groups resulting in hydrophilicity and thus, leading to stronger interactions with CNF.

**Figure 4:**
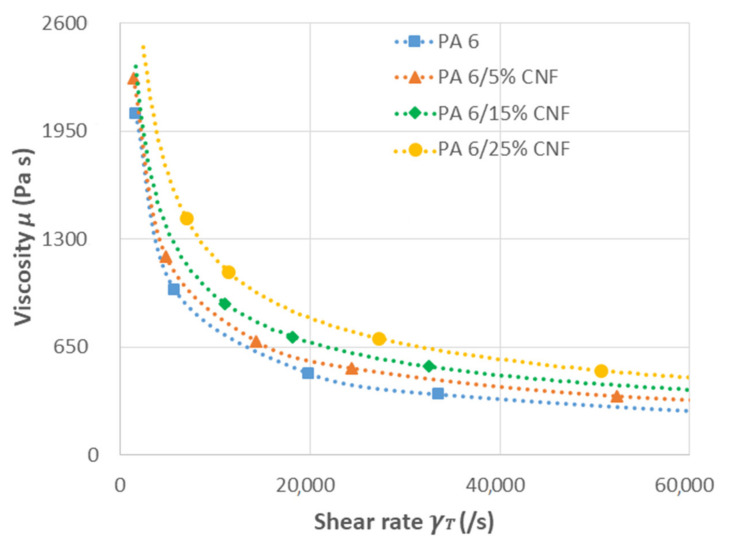
Rheological behavior of neat PA 6 and PA 6/CNF nanocomposites.

Capillary rheological properties are important to optimize the processing conditions and to know the rheological behavior of the nanocomposites with respect to the addition of CNF content. A rheogram representing variation of dynamic viscosity (*μ*) versus shear rate (*γ*_T_) can be obtained without having to use a sophisticated rheometer. We used an MFI type device to measure the melt flow indices of the samples with 5–6 different masses. By using different sets of masses, we were able to vary the shear stress. Subsequently, applying simple rheological equations, we traced a rheogram for the samples at 230 °C (Figure 4). The MFI values for the samples can be found in the Supplementary Materials. By studying the flow behavior through a capillary, relevant processing shear rates were characterized to confirm the process-ability of these nanocomposites. In addition, capillary rheological characterization by applied pressure closely resembles injection molding process [36]. All the samples displayed similar shear thinning behavior. PA 6/CNF nanocomposites behave as pseudoplastic fluids (non-Newtonian behavior) confirmed by decreasing viscosity with increasing shear rates. Moreover, pseudoplastic behavior is enhanced by the addition of CNF. For the nanocomposite samples, the viscosities increased with increase in CNF content with the highest viscosity seen for the 25 wt % formulation. At lower formulations (5 wt %), the CNF for the most part was like free particles and the nanocomposite melt had higher mobility. At higher formulations (15 and 25 wt %), the CNF disturbs the normal flow of nanocomposite melts and hinders the mobility causing high shear stresses, which is attributed to the hydrodynamic effect [37]. There was only a slight increase in viscosity for the nanocomposites samples when compared to neat PA 6, maybe due to the processing method employed and the aspect ratio of the CNF. This suggests the CNF was well dispersed and the presence of a percolating network [38]. Furthermore, rheological behavior is beneficial for process optimization and adjust processing conditions to improve the productivity.

**Figure 5:**
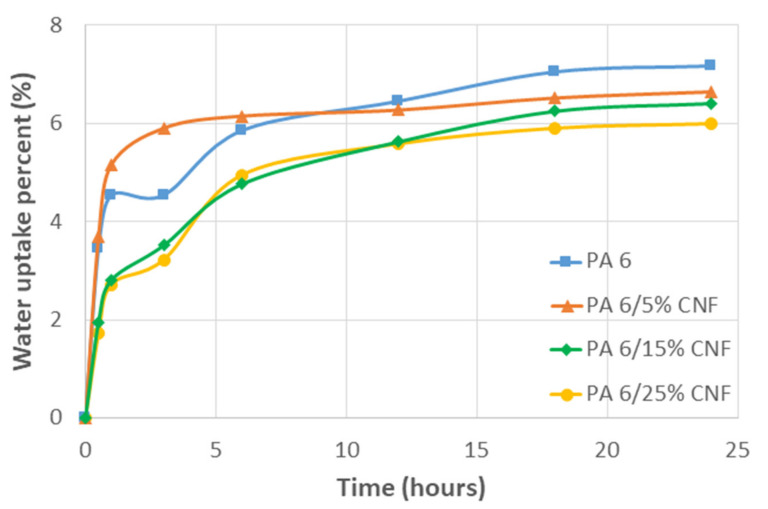
Water absorption percent versus time for neat PA 6 and PA 6/CNF nanocomposites at room temperature.

Further, water uptake study was carried out gravimetrically over a 24-h period. Mass measurements were noted periodically at 0 h, 0.5 h, 1 h, 3 h, 6 h, 12 h, 18 h and 24 h to determine the water absorption and associate it with the mechanical performance of the nanocomposite samples. In our case, two main interactions can be conjectured. The interaction between the water molecules and the amide groups of the PA 6. Then, the interaction of water molecules with the hydrophilic CNF. With the former dominating the absorption process in nanocomposite samples as the water uptake for nanocomposite samples decreased as function of CNF content but remained lower than the neat PA 6 (Figure 5). This is due to the strong hydrogen bonds between CNF and PA 6 along with the CNF–CNF interaction which are competing with water [39]. To study the influence of moisture on the mechanical properties, tensile samples were moisture conditioned for 48 h in a climatic chamber at 23 °C and relative humidity 50% prior to testing. From the Table 1, all the conditioned samples (48 h CC) showed reduced tensile modulus and strength. The reduction of modulus in the presence of moisture is common to most polyamides due to the plasticizing effect of water molecules on polyamides [40]. *T_g_* for moisture conditioned PA 6 can decrease to room temperature and below, as water molecules interfere with hydrogen bonds between the polymer chain increasing the chain mobility. The reduced *T_g_* causes a strong reduction of the amorphous modulus in the presence of moisture leading to decrease in the semicrystalline polymer [41]. Additionally, the reduction of modulus of matrix results in the modulus reduction of the nanocomposites. With increased mobility due to moisture conditioning, the yield stress also decreases and the deformation at break increases. Hence, moisture is a key factor affecting processing and final performance for PA 6/CNF nanocomposites.

Fick’s law was used to calculate diffusion coefficient *D* at lower times of immersion, i.e., when $M_{t}/M_{\infty }=0.5$. The diffusion coefficient for all the samples is summarized in Table 2. It was observed that diffusion coefficient decreased as the amount of CNF increased with 25 wt % formulation showing the least *D* value. The lower D values can be related to the lower mobility of PA 6 chains inhibited by CNF reinforcement. Nevertheless, hydrogen bonding between the fibers and the PA 6 matrix can also have a negative effect on the diffusion of water through the composite samples [42]. The $M_{\infty }$ values for nanocomposite samples were reached quicker when compared to neat PA 6 due to lower diffusion coefficients of the samples and the values remained below that of neat PA 6.

**Figure 6:**
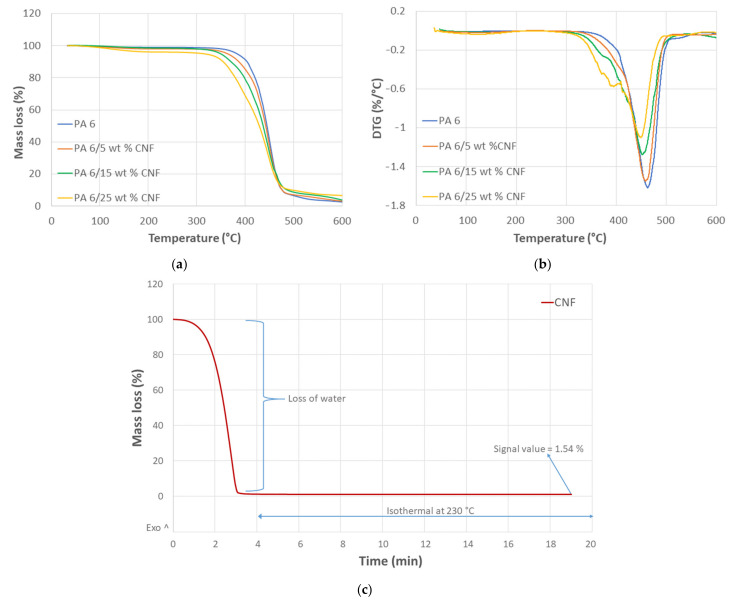
Thermograms, (**a**) TGA, (**b**) DTG for neat PA 6 and PA 6/CNF nanocomposites and (**c**) TGA of CNF gel at isothermal conditions at 230 °C.

Thermogravimetric analysis (TGA) was conducted to determine the thermal behavior of nanocomposites and compare it with the neat samples. Thermograms indicating TGA curves and DTG curves for all the samples are shown in Figure 6. TGA plot revealed the region of degradation (Figure 6a) and moreover, elucidated the temperature range for processing the nanocomposites. The initial mass loss within the temperature range of 60–110 °C is attributed to the evaporation of water from the samples. This loss of water is important because water acts as an auto accelerator in the degradation process [27]. For temperatures above 300 °C, degradation of cellulosic materials kicks off giving away combustible volatiles such as methanol, acetic acid acetaldehyde and propenal. These volatiles components increase the rate of decomposition in the nanocomposites [43]. For the nanocomposites, the thermal stability decreased mildly with an increase in CNF content. The CNF char residue for the samples lied between the range of 2–4%. From the first derivative of TG (Figure 6b), the DTG thermal bands were obtained at 460 °C for neat PA 6. In our case, a maximum temperature of 230 °C was used during the processing of nanocomposites, thus avoiding excessive thermal degradation of CNF.

The TGA was performed for the enzymatic CNF gel (1.5 wt % consistency) by heating the sample in an aluminum pan from 30 to 230 °C at a heating rate of 50 °C/min. At 4 min 230 °C was reached and the CNF was checked for thermal degradation at isothermal conditions for 15 min. The TGA curve for CNF at isothermal conditions (230 °C) is shown in Figure 6c. The initial drop in the TGA curve is due to the loss of water from the CNF gel. The remaining pure CNF does not experience any thermal degradation at the processing temperature. This indicates that the thermal stability of the CNF was not compromised at a processing temperature of 230 °C for this time.

**Figure 7:**
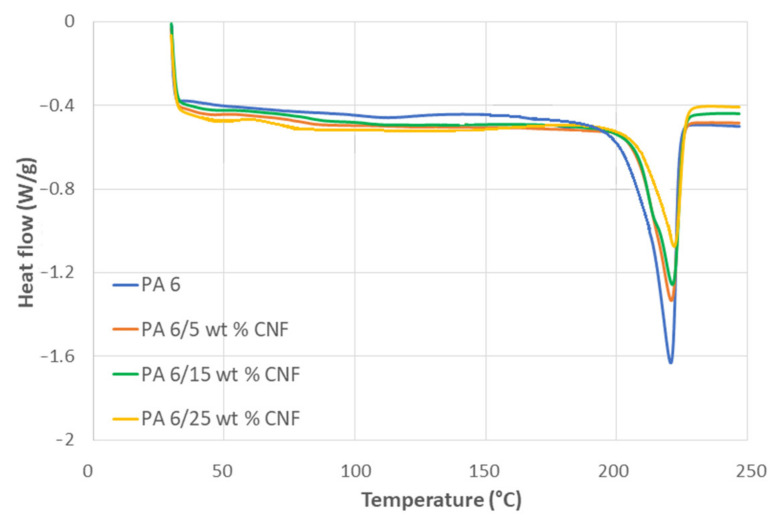
DSC thermogram, heating cycle for neat PA 6 and PA 6/CNF nanocomposites.

DSC analysis was conducted to recognize the effect of CNF content on the melting behavior and crystallinity of PA 6 matrix. Single, well defined endothermic peaks were obtained for all samples and the DSC thermogram for the heating cycle is shown in Figure 7. The values of glass transition temperature (*T_g_*), melting temperature (*T_m_*) and corresponding enthalpies (*∆H*) are summarized in Table 3, according to Equations (11) and (12) in the experimental section.

The DSC measurements indicate that the glass transition for the nanocomposite samples occurred at a slightly higher temperature when compared to neat PA 6. Additionally, the glass transition temperature increased with addition of CNF content. This was attributed to the strong interactions between polar amide groups of PA 6 and hydroxyl groups of CNF, thus reducing the segmental mobility of the nanocomposite structure [44]. The steady increase in *T_g_* also indicated that the CNF was well dispersed in the PA 6 matrix [45]. The melting point of the neat PA 6 from the endothermic melting peak was detected at 221.96 °C, which can be associated to the α-form crystals of PA 6 [46]. There was no significant influence on the melting point of the nanocomposite samples as the changes in Tm was minuscule. The *T_m_* for all samples were between 220 and 222 °C. The degree of crystallinity of the nanocomposite samples reduced with the addition of CNF. The crystallinity of PA 6 was 31.4%, while the crystallinity for the 25 wt % formulation was 24.8%. Identical decrease in crystallinity was observed for PA 6 nanocomposites containing microcrystalline cellulose and nanoclay [47,48]. The decrease in the crystallinity for nanocomposite samples is attributed to the vast surface on nanofillers, and the effect on the mobility of chains of polymer matrix. In some previous study, CNF acted as nucleating sites for crystallization [49]. The nucleating effect significantly contributes to the formation of transcrystalline layers around nanofillers thus, resulting in increase in crystallinity [50]. Nevertheless, the addition of nanofillers constrains the mobility of polymer chains hindering the crystal growth [45]. Thereby, decreasing the degree of crystallinity was the prevailing factor in our case.

**Figure 8:**
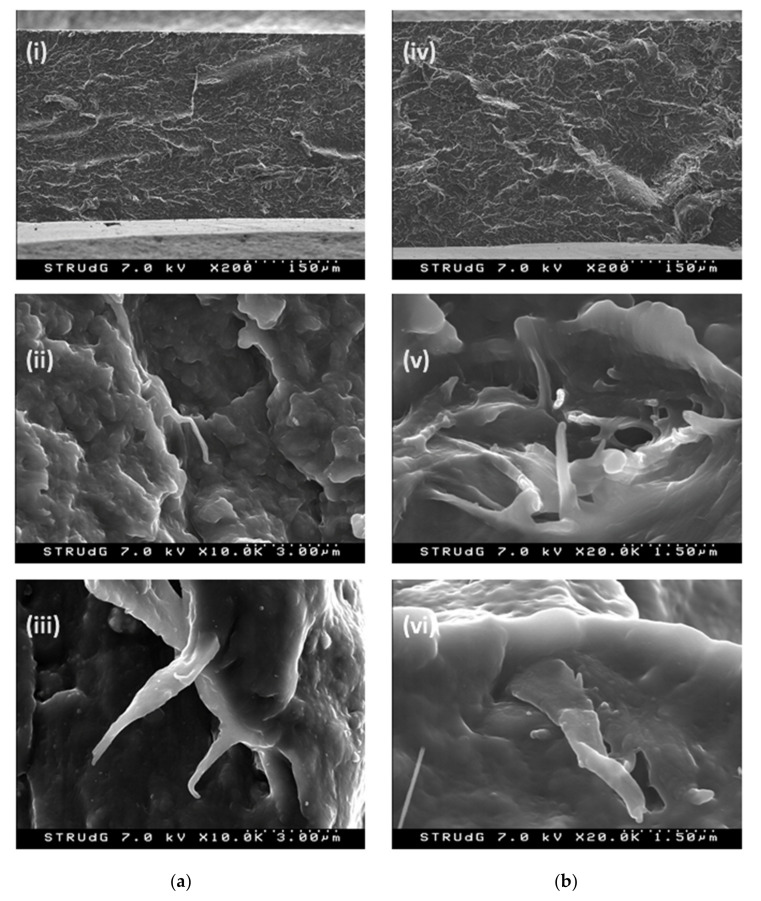
SEM micrographs of (**a**) 5 wt % and (**b**) 25 wt % PA/CNF nanocomposites.

The morphology of 5 wt % and 25 wt % formulations samples were observed under electron microscope. The SEM micrographs of the fractured surfaces for the nanocomposite samples are shown in Figure 8. The distribution of CNF appeared to be homogenous within the in-plane layers of the fractured samples with very few voids and no agglomerations in Figure 8(i),(iv). The fractured surfaces of the nanocomposite samples were almost identical to all formulations. The CNF show good adhesion to the PA 6 matrix and the interface bonding regions are observed in Figure 8(ii),(v). The morphology of fractured surfaces was formed under tension. Hence, the polymer strands had a fiber like shape, which were broken at some point during tensile elongation and were surrounded by CNF (Figure 8(v)). No major pull-out of the fibers was observed, but some broken fibers were found, which indicated suitable interface between PA 6 and CNF. Due to the hydrophilic nature of CNF and PA 6, the CNF demonstrated to be a suitable reinforcement to the polymer without problems of agglomeration. Under elongation, the different moduli of CNF and PA 6 created different deformation modes and the different strains were generated by the same load, which created stress concentration points at the interface of PA 6 and CNF. As this unmatched strain reached a magnitude of the interfacial adhesion between PA 6 and CNF, a sliding deformation occurs [31]. This sliding deformation is observed in Figure 8(vi). Moreover, the reinforcing effect was estimated by the effective stress transfer through interfacial adhesion, which was corroborated by the improved mechanical properties of the PA 6/CNF nanocomposites. Additionally, from the SEM micrographs, it was observed that the CNF integrity was unharmed, signifying no serious thermal degradation had taken place.

## 4. Conclusions

PA 6 with its material properties has become a hot commodity in the automobile industry. There is tremendous emphasis on enhancing the mechanical properties using natural nanofillers. CNF with its high specific strength, aspect ratio and compatibility with PA 6 became a tangible ecofriendly and sustainable choice of reinforcement. To produce CNF reinforced polymer composites at an industrial level, successful incorporation of CNF within the polymer matrix is critical. This study corroborated that premixing and compounding via a thermokinetic mixer, PA 6/CNF nanocomposites with good dispersion could be obtained. Minimizing the exposure time of CNF to high processing temperatures was key in obtaining composites with the preserved integrity of CNF. Three different formulations: 5, 15 and 25 wt % formulations of PA 6/CNF nanocomposites were produced. The mechanical properties were improved with the addition of CNF, with 25 wt % formulation showing the highest tensile values, in Young’s modulus and in tensile strength. Water uptake study indicated that the water uptake reduced with the addition of CNF due to the hydrogen bonds between the PA 6 and CNF, which hindered the diffusion of water molecules. DSC analysis revealed that the degree of crystallinity reduced for the nanocomposites as the addition of CNF constrained the polymer chain hindering crystal growth. TGA expectedly implied that the thermal stability of nanocomposites reduced slightly for the nanocomposites. The capillary rheological study indicated that the dynamic viscosity increased with the addition of CNF. SEM micrographs showed homogenous interface morphology of fractured samples with hardly any fiber pull-out.

Further, producing samples with injection molding and optimizing processing methods will further increase the scalability of CNF/polymer composites on an industrial level. Ultimately, this study provides insightful prospects on sustainable materials and developing high performance nanocellulose reinforced polymer composites with high fiber weight fractions.

## Figures and Tables

**Table 1:** Summary of tensile test results of dry and 48 h conditioned samples for neat PA 6 and all formulations of nanocomposites.

CNF Content (wt %)	Mean Thickness (mm)		Ultimate Strength (MPa)		Elastic Modulus (GPa)		Strain at Break (%)	
	Dry	48 h CC	Dry	48 h CC	Dry	48 h CC	Dry	48 h CC
Neat PA 6	0.46 ± 0.05	0.48 ± 0.03	50.8 ± 0.62	47.8 ± 1.1	2.2 ± 0.1	2.1 ± 0.3	9.8 ± 5.4	10.4 ± 5.6
5	0.35 ± 0.05	0.29 ± 0.06	56.5 ± 1.1	53.6 ± 0.7	3.9 ± 0.7	3.5 ± 0.7	2.3 ± 0.6	2.5 ± 0.5
15	0.30 ± 0.04	0.25 ± 0.08	65.6 ± 1.5	61.7 ± 2.1	4.8 ± 0.6	4.3 ± 0.3	1.6 ± 0.4	1.8 ± 0.2
25	0.25 ± 0.04	0.31 ± 0.03	77.7 ± 2.01	73.2 ± 2.5	5.6 ± 0.1	5.4 ± 0.4	1.2 ± 0.3	1.3 ± 0.1

**Table 2:** Diffusion coefficient at 23 °C for PA 6/CNF nanocomposites.

CNF Content (wt %)	$M_{\infty }\;(\%)$	*D* × 10^−9^ (m^2^ s^−1^)
Neat PA 6	7.2	6.5
5	6.8	3.2
15	6.6	0.6
25	6.1	0.5

**Table 3:** Thermal properties of neat PA 6 and PA 6/CNF nanocomposites.

CNF Content (wt %)	*T_g_* (°C)	*T_m_* (°C)	Δ*H_polymer_* (J/g)	*χ_c_* (%)
Neat PA 6	51.6	221.96	72.1	31.4
5	53.5	221.32	60.49	26.3
15	54.7	220.91	59.81	26.0
25	55.4	220.69	57.12	24.8

## Data Availability

The data presented in this study are available on request from the corresponding author.

## Supplementary Materials

The following are available online at https://www.mdpi.com/article/10.3390/polym13091495/s1, Table S1: melt flow indices (MFI) and melt volume flow rate (MVR) for neat PA 6 and PA 6/CNF nanocomposites at different masses.
