# Supplementary material for: High Performance PA 6/Cellulose Nanocomposites in the Interest of Industrial Scale Melt Processing
Source: Polymers (Basel). 2021 May 6;13(9):1495. doi: 10.3390/polym13091495 (PMC8124158; doi:10.3390/polym13091495)
Supplement: Supplementary file 1 [file polymers-13-01495-s001.zip › polymers-1210521-supplementary.pdf]

polymers

MDPI

Article

High Performance PA 6/Cellulose Nanocomposites in the Interest of Industrial Scale Melt Processing

Pruthvi K Sridhara and Fabiola Vilaseca *

Advanced Biomaterials and Nanotechnology, Department of Chemical Engineering, University of Girona, 17003 Girona, Spain; pruthvi.sridhara@udg.edu
* Correspondence: fabiola.vilaseca@udg.edu; Tel.: +34-667-292-597

Citation: Sridhara, P.K.; Vilaseca, F. High Performance PA 6/Cellulose Nanocomposites in the Interest of Industrial Scale Melt Processing. *Polymers* **2021**, *13*, 1495. https://doi.org/10.3390/polym13091495

Academic Editor: Giulia Fredi

Received: 20 April 2021
Accepted: 3 May 2021
Published: 6 May 2021

Publisher's Note: MDPI stays neutral with regard to jurisdictional claims in published maps and institutional affiliations.

Copyright: © 2021 by the authors. Licensee MDPI, Basel, Switzerland. This article is an open access article distributed under the terms and conditions of the Creative Commons Attribution (CC BY) license (http://creativecommons.org/licenses/by/4.0/).

Supplementary Materials

Table S1. Melt flow indices (MFI) and melt volume flow rate (MVR) for neat PA 6 and PA 6/CNF nanocomposites at different masses.

CNF Content (wt.%)		Mass						
		**M1 (1.2 kg)**	**M2 (2.16 kg)**	**M3 (3.8 kg)**	**M4 (5 kg)**	**M5 (7.16 kg)**	**M6 (10 kg)**	**M7 (12.16 kg)**
**PA 6**	MFI (g/10min)	4.74	9.07	16.95	22	34.63	55.33	-
	MVR (m^3/s)	0.0069	0.013	0.025	0.032	0.051	0.08	-
5	MFI (g/10min)	4.66	8.76	14.85	19.38	28.46	39.76	-
	MVR (m^3/s)	0.0066	0.013	0.022	0.028	0.041	0.058	-
15	MFI (g/10min)	-	-	13.48	17.08	22.63	33.53	41.98
	MVR (m^3/s)	-	-	0.019	0.024	0.032	0.047	0.059
25	MFI (g/10min)	-	-	10.64	13.68	21.12	28.76	34.34
	MVR (m^3/s)	-	-	0.015	0.019	0.029	0.04	0.047
